# Teaching staff as gatekeepers for student mental health in higher education: findings from an exploratory sequential mixed-methods study on mental health promotion, psychological crises, and stigma

**DOI:** 10.1186/s13033-026-00738-5

**Published:** 2026-09-18

**Authors:** Maria Koschig, Michéle Müller, Steffi G. Riedel-Heller, Ines Conrad

**Affiliations:** Institute of Social Medicine, Occupational Medicine and Public Health, University of Leipzig, Philipp-Rosenthal-Straße 55, Leipzig, 04103 Germany

**Keywords:** Mental health literacy, Stigma, Higher education, University teaching staff, Help-seeking, Mixed-methods research, Psychological crises

## Abstract

**Background:**

Mental health problems among university students are highly prevalent, while stigma and limited help-seeking remain major barriers to support utilization. Teaching staff can act as important gatekeepers who recognise distress, encourage help-seeking, and contribute to psychologically supportive learning environments. However, their role in student mental health promotion remains insufficiently understood.

**Methods:**

This exploratory sequential mixed-methods study combined a qualitative focus group involving five students from two German universities and an anonymous online survey of teaching staff from four German higher education institutions (*n* = 187). Insights from the focus group informed the development and content of the subsequent survey of teaching staff. Qualitative data were analysed using structured content analysis according to Kuckartz. Quantitative analyses included descriptive statistics and assessment of stigma-related attitudes using the Self-Stigma of Mental Illness Scale Short Form (SSMIS-SF).

**Results:**

The qualitative findings revealed four interrelated areas of expectation: psychologically sensitive communication, mental health literacy among teaching staff, accessible institutional support structures, and a university culture that normalises help-seeking. Among teaching staff, only 30.9% considered student mental health promotion part of their formal professional role, while 45.8% reported uncertainty when interacting with students experiencing psychological crises. Qualitative findings indicated substantial variation in perceived preparedness and support practices. Teaching staff primarily described recognising distress, offering initial support, and referring students to professional services. Personal endorsement of stigmatizing beliefs was low, whereas perceived public stigma was significantly higher (mean difference = 12.42, 95% CI 11.02–13.83; *t*(131) = 17.51, *p* < .001; Cohen’s *d* = 1.52, 95% CI 1.27–1.77).

**Conclusions:**

Teaching staff may represent important gatekeepers for student mental health. However, unclear role expectations, limited institutional guidance, and insufficient training may restrict their capacity to support students effectively. Universities should strengthen staff training, establish clear referral pathways, and implement institution-wide mental health strategies.

**Supplementary Information:**

The online version contains supplementary material available at https://doi.org/10.1186/s13033-026-00738-5.

## Background

Mental health problems among university students have become an increasing public health concern worldwide. Overall, psychological distress is highly prevalent in student populations globally, with particularly elevated rates observed during the COVID-19 pandemic and among medical students [1–4]. Higher education students, especially undergraduate, report elevated levels of psychological distress, including anxiety, depression, stress-related symptoms, crisis experiences [5–7], and insomnia symptoms [8]. A large systematic review and meta-analysis of Chinese medical students estimated pooled prevalence rates of 29% for depression and 18% for anxiety [3]. Similarly, a regional systematic review across ASEAN countries reported prevalence estimates of 29.4% for depression and 42.4% for anxiety [4], highlighting substantial cross-national variability. These mental health problems may negatively affect academic performance, social participation, and overall well-being [9]. Despite growing awareness of student mental health needs, help-seeking behaviour among students remains limited, and many affected individuals do not access professional support services [10–12]. Studies suggest that it is precisely the students under the most stress who are less likely to seek help [13]. Mental health-related stigma represents one of the most important barriers to disclosure and help-seeking within higher education contexts [14, 15].

Research has demonstrated that stigma associated with mental illness operates on multiple levels, including public stigma, self-stigma, structural stigma, courtesy stigma, and provider-related stigma [14, 15]. Within university settings, stigma may become embedded in institutional cultures, communication practices, and interpersonal interactions. Students experiencing psychological distress frequently report fears of discrimination, concerns regarding academic disadvantages, and reluctance to disclose mental health problems to peers or teaching staff [15, 16]. In addition, self-stigma and stigma by association may further reduce willingness to seek support or communicate openly about psychological crises [17, 18].

Against this background, universities are increasingly recognised as important settings for mental health promotion and stigma reduction. Therefore, mental health is considered to be the result of institutional, social, and curricular conditions, rather than an individual problem [19, 20]. Existing research suggests that psychologically supportive learning environments, visible support structures, and destigmatising communication may contribute to improved mental health literacy and increased help-seeking among students [21, 22]. In particular, educational approaches combining psychoeducation with direct interpersonal contact involving individuals with lived experience of mental illness have shown promising effects in reducing stigma and increasing empathy [16, 21, 22].

Within higher education institutions, teaching staff may represent important gatekeepers in relation to student mental health. Gatekeeper approaches describe individuals in everyday social or institutional roles who are able to recognise signs of psychological distress, initiate supportive conversations, and facilitate referral to professional services [16, 21]. Beyond their gatekeeper function, teaching staff may also influence students through role modelling and observational learning processes. Social learning perspectives suggest that students are shaped not only by formal instruction but also by observing the attitudes, communication styles, coping behaviours, and interpersonal interactions demonstrated by influential others. In higher education settings, teaching staff may therefore contribute to the formation of students’ beliefs and norms regarding mental health, help-seeking, and psychological distress, often through implicit and unintentional processes [23–25]. By modelling openness, supportive communication, and non-stigmatizing responses to mental health-related issues, teaching staff may foster psychologically safer learning environments and contribute to the normalisation of help-seeking behaviours. At the same time, several barriers may limit teaching staff’s ability to fulfil such functions. Existing research suggests that many university staff members report uncertainty regarding professional responsibilities, limited knowledge of referral structures, and insufficient training in responding to psychological crises among students [16, 21]. Furthermore, stigma-related attitudes and perceived social norms surrounding mental illness may influence communication behaviours, disclosure processes, and students’ willingness to seek support [14, 15]. Although previous studies have examined student mental health, anti-stigma interventions, and mental health literacy within higher education, comparatively little attention has been paid to how teaching staff themselves perceive their role, competencies, and responsibilities in supporting student mental health. The present exploratory sequential mixed-methods study therefore investigated how students and teaching staff perceive the role of teaching staff in relation to mental health promotion, psychological crises, and help-seeking within higher education. The qualitative phase explored students’ expectations and experiences and informed the development of the subsequent quantitative survey among teaching staff. By integrating student perspectives with quantitative and qualitative data from teaching staff, the study aimed to generate a more comprehensive understanding of role perceptions, support practices, and stigma-related processes within higher education institutions. The study addressed the following research questions:

1. What **expectations** and support needs do university students report regarding teaching staff and university institutions in relation to mental health, psychological crises, and help-seeking within higher education settings?
2. How do university teaching staff perceive their own **competencies**, preparedness, and support needs when dealing with students experiencing psychological distress or psychological crises?
3. To what extent do university teaching staff perceive the promotion of student mental health and support for psychologically distressed students as part of their **professional role** within higher education?
4. To what extent may **stigma-related attitudes** and perceived public stigma among university teaching staff influence supportive interactions with students and the potential gatekeeper role of teaching staff in higher education?

## Methods

### Study design

The present study employed an exploratory sequential mixed-methods design integrating qualitative and quantitative approaches to investigate the role of teaching staff in relation to student mental health, psychological crises, and stigma-related processes within higher education settings following the methodological framework described by Creswell and Plano Clark [26]. The design was chosen because little is known about students’ perspectives on the role of teaching staff in supporting mental health within German higher education and no suitable survey instrument addressed the research questions. The initial qualitative phase therefore explored students’ perspectives and generated themes that informed the subsequent survey of teaching staff.

The study began with a qualitative student focus group, which enabled participants to discuss shared experiences and collectively identify issues that may not emerge through standardised questionnaires. The discussion explored students’ experiences with psychological distress, expectations regarding teaching staff, barriers to help-seeking, preferred communication and referral practices, and institutional facilitators and barriers to mental health support.

The findings from this exploratory phase informed the conceptual orientation, wording, and thematic priorities of the subsequent quantitative online survey. Study-specific items were developed iteratively by combining themes and support needs identified in the focus group with constructs from the literature on mental health promotion, stigma, help-seeking, and the role of teaching staff. The preliminary questionnaire was subsequently reviewed together with experts involved in the prevention programme “Studying and Staying Mentally Healthy: Universities in Dialogue” (Totally Human e. V.) and refined regarding wording, content, and practical relevance. Integration occurred at two stages. First (“building integration”), qualitative findings informed survey domains, item wording, and the prioritisation of student-relevant topics. Second (“joint interpretation”), qualitative and quantitative findings were considered together to identify convergence and divergence between students’ expectations and teaching staff’s self-reported attitudes, responsibilities, preparedness, support practices, and institutional support needs. Thus, the qualitative phase fulfilled a developmental function during instrument construction and a complementary function during interpretation.

### Qualitative phase: student focus group

The qualitative phase consisted of a semi-structured focus group discussion conducted in December 2022. The focus group was designed as an exploratory component of the mixed-methods study and addressed experiences with psychological distress during university studies, expectations towards teaching staff and universities, perceived barriers to help-seeking, and views regarding supportive teaching practices and stigma within higher education. Participants also discussed the perceived responsibilities of teaching staff, preferred approaches to students experiencing psychological distress, referral options, and institutional conditions affecting support. The semi-structured format allowed participants to elaborate on emerging topics and build on each other’s experiences.

The focus group was conducted in person, audio-recorded, and transcribed verbatim prior to analysis. Qualitative data were analysed using structured qualitative content analysis according to Kuckartz [27]. Initial categories were developed deductively based on the research questions and subsequently refined and expanded inductively during the coding process. Coding focused on recurring themes related to support expectations, stigma, communication practices, and institutional mental health structures, particularly those that could be translated into survey domains or study-specific items.

The analysis was supported by MAXQDA software. Two researchers independently coded the material. The AI Assist function integrated within MAXQDA facilitated the identification and structuring of recurring themes for inductive categories. The use of AI Assist was restricted to supporting the organisation and exploration of qualitative data. The tool did not perform autonomous analysis, interpretation, or validation of findings. Responsibility for all analytical decisions remained entirely with the research team. Coding discrepancies and category definitions were discussed iteratively and resolved through consensus among the researchers. Representative quotations were translated from German into English for reporting purposes and double-checked using DeepL. Given the exploratory character of the qualitative phase, the focus group was not intended to achieve representativeness or thematic saturation. Instead, its purpose was to identify salient themes, language, and perspectives relevant to the students’ expectations of teaching staff and to inform the subsequent survey. Consequently, the findings are hypothesis-generating rather than representative of the broader student population. The resulting thematic framework was reviewed by the research team before being translated into the questionnaire structure and content.

### Quantitative phase: online survey among teaching staff

The second study phase consisted of an anonymous online survey conducted among university teaching staff conducted in 2024. The questionnaire included items assessing perceived professional responsibilities related to student mental health, confidence in dealing with students experiencing psychological crises, support practices, perceived support needs, and stigma-related attitudes towards mental illness. In addition to closed-ended items, the survey contained several open-ended questions allowing participants to describe their experiences, support practices, and perceived institutional challenges in greater detail.

### Participants

**Fig. 1:**
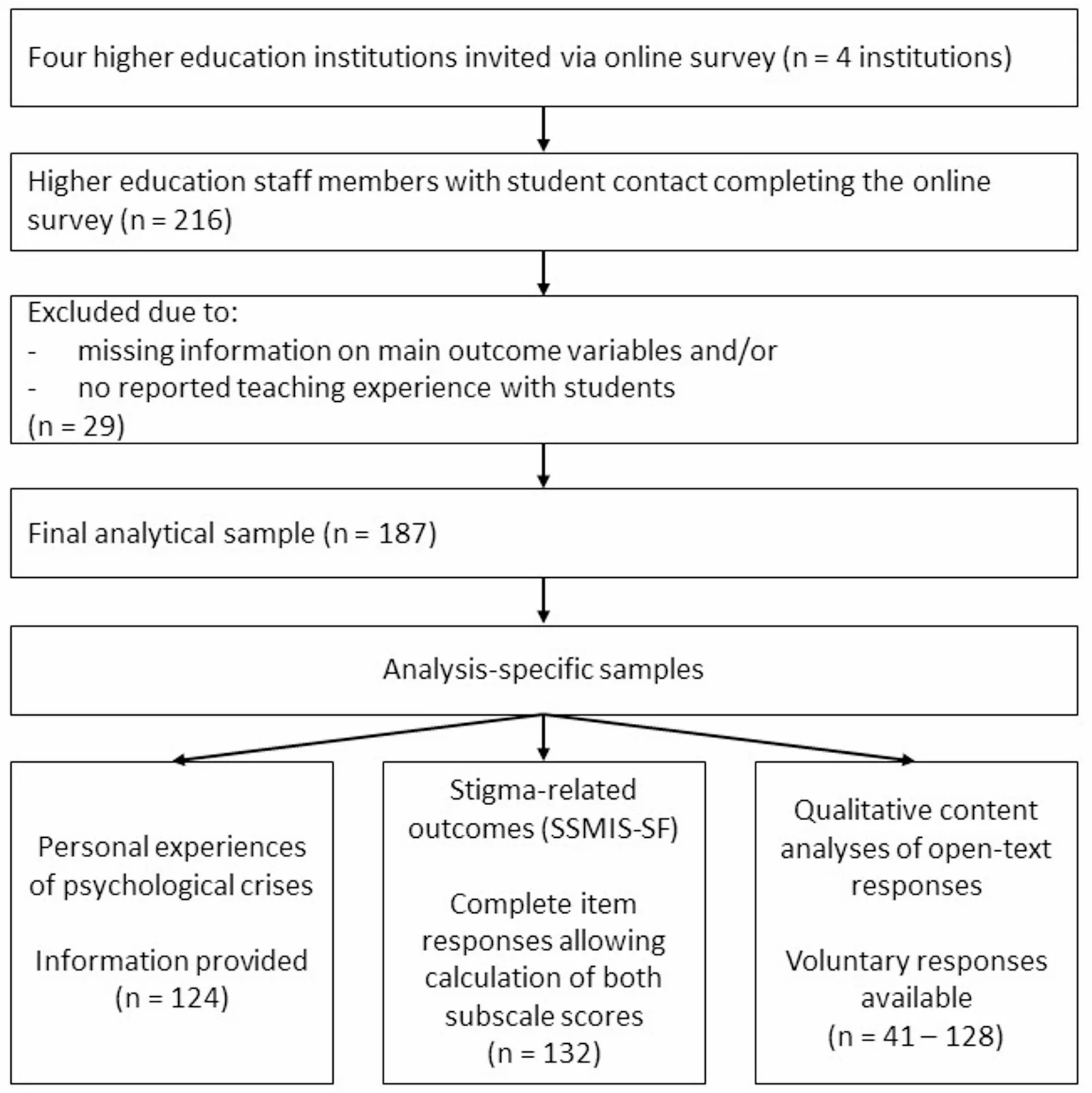
Participant flow and analysis-specific sample sizes resulting from item-level missing data and optional questionnaire sections

The *qualitative phase* included students from two German universities recruited via purposive sampling. Inclusion criteria were enrolment at a German higher education institution and willingness to participate in a discussion on student mental health and university support structures. Participants were recruited through informal peer-based recruitment. Researchers contacted peers within their networks, who were informed about the study and encouraged to share the invitation with potentially interested students. Five students subsequently participated in the focus group. The five participants represented different academic disciplines. The *quantitative phase* comprised an anonymous online survey among teaching staff from four German higher education institutions. A total of 216 individuals accessed the survey. After exclusion of incomplete questionnaires and respondents without teaching-related activities, the final analytical sample consisted of 187 participants, recruited through institutional mailing lists and internal communication channels. Figure 1 provides an overview of the participant flow and illustrates the analysis-specific sample sizes resulting from item-level missing data and the voluntary completion of additional questions.

### Measures

Stigma-related attitudes were assessed using the Awareness and Agreement subscales of the Self-Stigma of Mental Illness Scale Short Form (SSMIS-SF) [28, 29]. The Awareness subscale measures perceptions of public stigma towards individuals with mental illness, whereas the Agreement subscale assesses personal endorsement of stigma-related stereotypes. Responses were rated on a 5-point Likert scale ranging from 0 (“strongly disagree”) to 4 (“strongly agree”). Higher scores indicate higher levels of perceived public stigma or personal agreement with stigmatising beliefs, respectively. The SSMIS-SF exhibited acceptable to high internal consistency across its subscales, with Cronbach’s alpha coefficients ranging from 0.65 to 0.87 [29].

In addition to the validated SSMIS-SF, the questionnaire included study-specific items assessing perceived professional responsibilities, confidence in responding to students experiencing psychological crises, communication and referral practices, institutional support structures, training needs, and attitudes towards mental health promotion within higher education. These items were developed specifically for the present study because no existing instrument adequately addressed the research questions and institutional context. As described above, their development was informed by the qualitative findings, relevant literature, and expert consultation. Sociodemographic variables included age, gender, duration of employment in higher education, weekly teaching workload, and university affiliation. The questionnaire is provided as Additional file 1 (Supplementary Material).

### Statistical analysis

Quantitative analyses were conducted using Statistical Package for the Social Sciences (SPSS) software version 29. Descriptive statistics included frequencies (*n*), percentages, means (*M*), standard deviations (*SD*), and ranges.

Group differences between participating universities regarding stigma-related attitudes were analysed using one-way analyses of variance (ANOVA). Effect sizes were reported using eta-squared (η²). Statistical significance was defined as *p* < .05. The SSMIS-SF subscale scores were computed as summed scores, with higher values indicating greater stigma-related endorsement. No imputation of missing values was performed. Missing responses were handled on an analysis-specific basis by excluding only cases with missing information required for the respective analysis (available-case analysis). This approach maximised the available sample size while avoiding unnecessary case exclusion. Non-response analyses showed no significant differences in age, sex, and years of employment in higher education. The varying analysis-specific sample sizes resulting from item-level missing data are presented in the participant flow diagram (Fig. 1) and reported in the respective tables where applicable. A paired-samples t-test was conducted to examine differences between SSMIS-SF awareness and agreement scores. Normality of difference scores was assessed using the Shapiro–Wilk test and was not violated (*W* = 0.983, *p* = .101).

Qualitative free-text responses from the online survey were additionally analysed using structured qualitative content analysis according to Kuckartz [27]. Responses were coded inductively, and thematic categories were developed iteratively across the dataset. Coding was conducted collaboratively by two researchers using consensus-based category refinement. Representative quotations (“anchor examples”) were selected to illustrate major categories identified during the analysis.

### Ethical considerations

#### Ethical approval

for the study was obtained from the responsible institutional ethics committee prior to data collection. Participation in both study phases was voluntary. All participants received written information regarding the study aims, data protection procedures, and confidentiality prior to participation. Informed consent was obtained from all participants before participation in the focus group discussion and online survey. All data were analysed anonymously and stored in accordance with applicable data protection regulations.

## Results

### Student expectations and needs regarding teaching staff

The qualitative focus group analysis identified several recurring themes regarding students’ expectations towards teaching staff in relation to mental health and psychological crises within higher education settings. Students emphasised the importance of open and psychologically sensitive communication by teaching staff. Participants described a preference for teaching staff who communicate openly about stress, psychological strain, and coping with overload. In addition, students highlighted the relevance of approachable and empathetic communication styles in facilitating supportive interactions within academic contexts.

Participants further reported expectations that teaching staff should possess basic knowledge regarding mental health problems, psychological crises, and available university support structures. Teaching staff were frequently described as important first points of contact who should be able to recognise signs of psychological distress and provide guidance towards professional support services. One participant explained: “*It would help if lecturers knew where to refer students. They do not need to become therapists*,* but they should know where support is available.*” Another recurring theme concerned the need for training and qualification measures for teaching staff. Students stated that teaching staff should receive education on mental health, supportive communication, and institutional procedures for dealing with students experiencing psychological distress. Participants additionally referred to insufficient transparency regarding compensation measures for disadvantages related to illness or disability.

Students also described the importance of supportive teaching cultures characterised by respectful communication, reduced hierarchy, and opportunities for interpersonal exchange. One student stated: “*Mental health should not depend on individual lecturers. It should be visible throughout the university.*” Smaller seminar formats and interactive teaching approaches were perceived as facilitating communication and psychological well-being. At the institutional level, participants emphasised the need for visible and low-threshold support systems for students experiencing psychological crises. Existing support structures were described as difficult to access or insufficiently transparent. Students additionally referred to the importance of preventive approaches, including stress management, self-organisation, and mental health-related educational content integrated into university curricula.

**Fig. 2:**
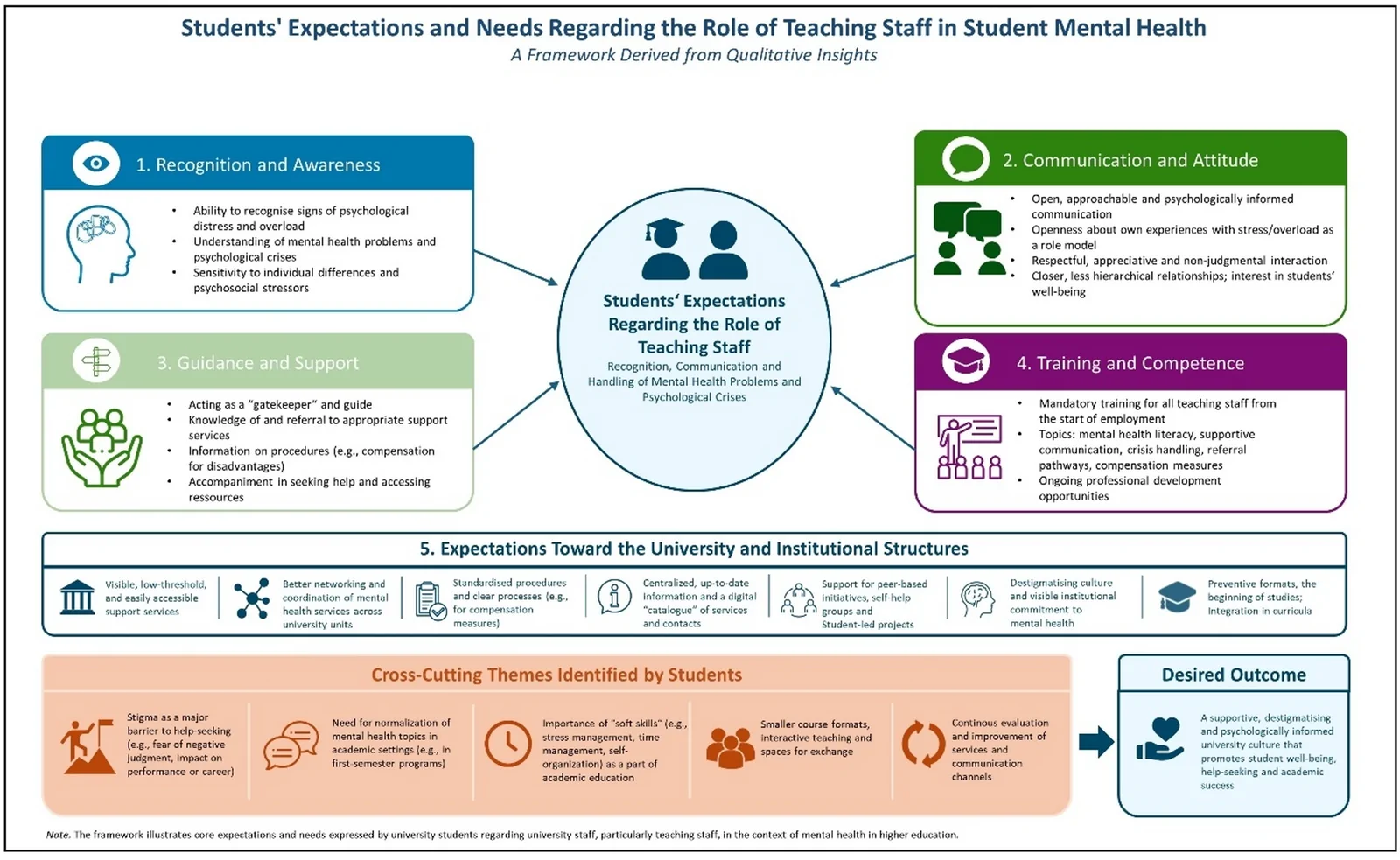
Conceptual overview of students’ expectations regarding teaching staff and institutional support for student mental health derived from the qualitative content analysis

Several comments indicated stigma-related concerns as barriers to help-seeking within higher education. As one participant noted: “*If you disclose a mental health problem*,* you never know whether it will be interpreted as a lack of resilience or ability.*” Psychological distress and mental illness were associated with fears regarding academic disadvantages and future career consequences. Figure 2 summarises the central themes identified in the qualitative analysis.

### Sample characteristics of teaching staff

A total of 216 individuals accessed the online survey. After exclusion of incomplete questionnaires and respondents without teaching-related activities, the final analytical sample consisted of 187 teaching staff members involved in teaching activities. The mean age of participants was 44.47 years (*SD* = 11.39; range: 25–66 years). The average duration of employment in higher education was 14.07 years (*SD* = 9.88; range: 0.5–40 years). Data on weekly teaching workload were available for 181 participants, with a mean teaching load of 7.99 semester contact hours per week (*SD* = 6.37; range: 0.5–40). Regarding gender distribution, 43.85% of participants identified as male (*n* = 82) and 56.15% as female (*n* = 106). Among the 152 participants who responded to the item assessing perceived responsibility for promoting student mental health, 30.92% (*n* = 47) reported that they considered this responsibility part of their professional role, whereas 69.08% (*n* = 105) did not. Sample characteristics are presented in Table 1.

**Table 1:** Characteristics of teaching staff and perceived role-related responsibilities in higher education institutions (*n* = 187)

Variables	Category	*n*	%	M (SD)	Min	Max
Age (years)	—	—	—	44.47 (11.39)	25.00	66.00
Employment duration in higher education (years)	—	—	—	14.07 (9.88)	0.50	40.00
Weekly teaching load (semester hours per week), *n* = 181	—	—	—	7.99 (6.37)	0.50	40.00
Gender	Male	82	43.85	—	—	—
	Female	105	56.15	—	—	—
Mental health promotion responsibilities linked to academic role, *n* = 152	Yes	47	30.92	—	—	—
	No	105	69.08	—	—	—

Values are presented as mean (M), standard deviation (SD), minimum (Min), maximum (Max), absolute numbers (n), and percentages (%)

### Perceived professional role in student mental health promotion

Open-ended responses from participants who perceived student mental health promotion as part of their professional role and described it in more detail were analysed qualitatively (*n* = 41 statements). Six main categories were identified. The largest category referred to *individual counselling*,* mentoring*,* and crisis support*. Participants described providing emotional support, responding to crisis situations, and accompanying students during periods of psychological strain. As one participant explained, *“As a study program coordinator*,* I am often the first point of contact in all life situations and provide counselling in difficult circumstances*,* including psychological crises*,* and refer students to appropriate services.”*

A second category concerned *psychoeducation*,* mental health literacy*,* and prevention*. Respondents described integrating reflection exercises, stress-management strategies, and self-care-related topics into teaching activities. Additional responses referred to *structural teaching design and workload regulation*, including adaptations of teaching formats, supervision practices, and examination procedures to reduce academic overload.

Participants also described *signposting and referral practices*, including directing students toward psychosocial counselling services and institutional support structures. Another category reflected *role modelling and destigmatisation practices*, including empathic communication, normalisation of stress, and the creation of supportive learning environments. Finally, some respondents described *institutionalised mental health-related responsibilities* linked to formal administrative or coordination roles.

**Fig. 3:**
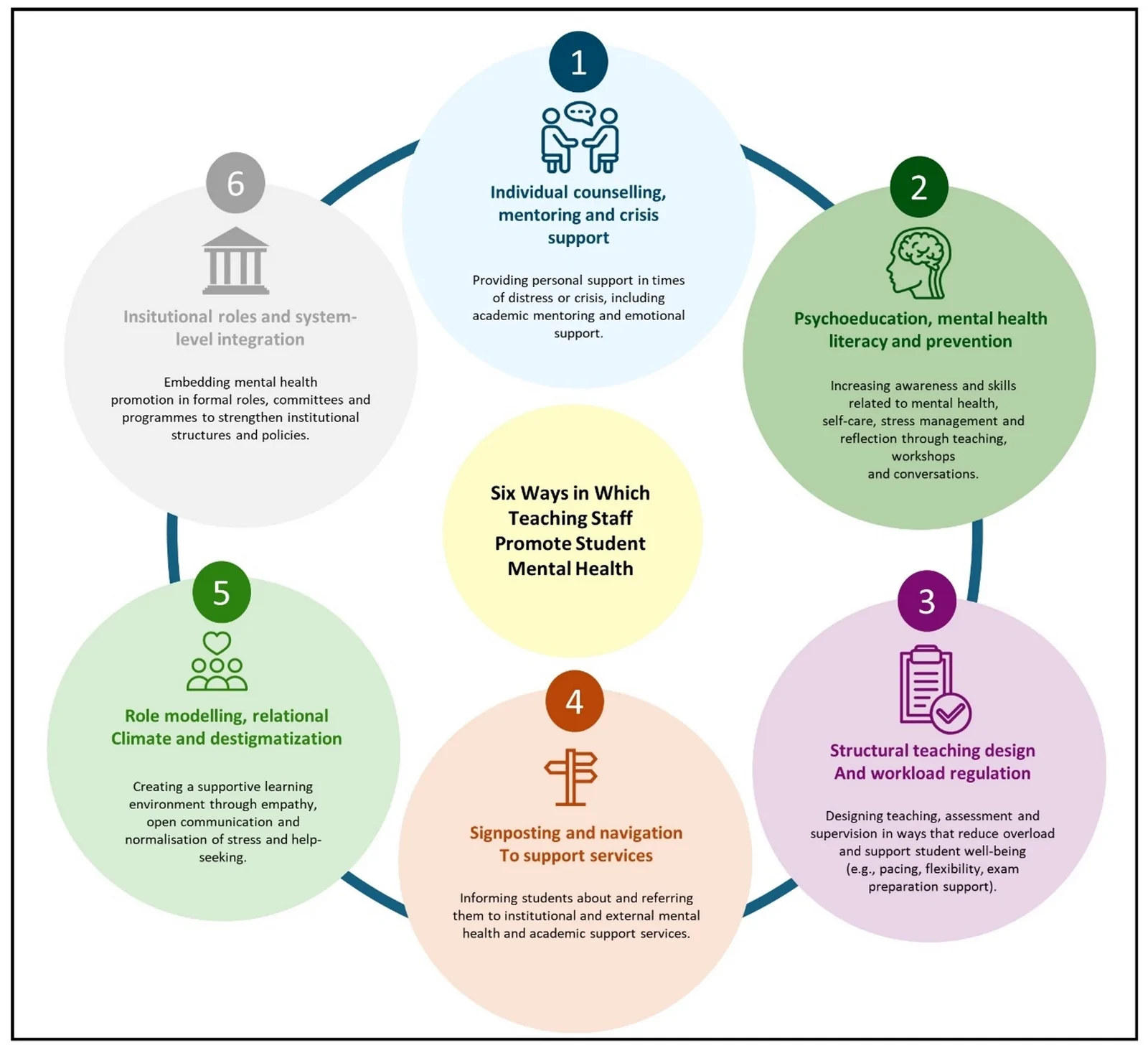
Categories of teaching staff–reported activities supporting student mental health, derived from qualitative content analysis of open-ended survey responses

Table 2 provides an overview of the identified categories and illustrative quotations. Figure 3 illustrates the thematic distribution of identified role-related activities.

**Table 2:** Categories of academic staff contributions to student mental health promotion (*n* = 41)

Category	Description	*n*	Anchor example
1. Individual counselling, mentoring and crisis support	Direct interpersonal support for students experiencing psychological distress, including crisis response, emotional support, and academic mentoring in high-stress situations.	15	*“As a study program coordinator*,* I am often the first point of contact in all life situations and provide counselling in difficult circumstances*,* including psychological crises*,* and refer students to appropriate services.”*
2. Psychoeducation, mental health literacy and prevention	Activities aimed at increasing students’ awareness of mental health, stress management, self-care, and psychological processes through teaching, workshops, or structured reflection.	10	*“I offer a voluntary self-care workshop and explicitly integrate reflection and workload balance into my seminars*,* including structured opportunities for self-reflection during internships.”*
3. Structural teaching design and workload regulation	Adjustments in teaching, assessment, and supervision to reduce student overload, including pacing, exam preparation support, and workload-sensitive course design.	8	*“Learning tasks are designed in a way that students are not overwhelmed; I actively reduce pressure in exam preparation and supervision contexts.”*
4. Signposting and navigation to support services	Guiding students towards institutional or external mental health and academic support systems, including referrals and information provision.	7	*“I refer students to existing counselling and support services at the university whenever I identify a need for additional assistance.”*
5. Role modelling, relational climate and destigmatisation	Creating supportive learning environments through empathic communication, normalisation of stress, and modelling healthy coping and professional behavior.	6	*“As a professor*,* I try to maintain an open ear and eye*,* recognize who needs help*,* and create a learning environment where mistakes are seen as part of learning.”*
6. Institutional roles and system-level mental health integration	Mental health promotion embedded in formal administrative roles, governance structures, and institutional programs (e.g., coordination, equality, disability services, SGM projects).	5	*“As head of a study program*,* I also provide coaching and serve as a key contact person for students with psychological burdens within institutional structures.”*

The table presents results of a qualitative content analysis of open-ended survey responses from academic staff across four higher education institutions (*n* = 41 statements). Each statement was treated as a single unit of analysis. Categories were developed inductively using an iterative coding process. Frequencies (n) indicate the number of statements assigned to each category, and exemplary quotations illustrate typical manifestations of each theme

### Perceived confidence in dealing with students experiencing psychological crises

Qualitative content analysis was conducted for responses to the question assessing perceived confidence in dealing with students experiencing psychological crises. Empty responses were excluded from the analysis. A total of 120 valid responses were included. Four overarching categories describing different levels of perceived confidence were identified. Twenty-one participants (17.5%) were categorised as very insecure or overwhelmed. These respondents frequently referred to lack of training, uncertainty regarding appropriate responses, and insufficient institutional resources. One participant summarised this uncertainty by stating: *“Very insecure*,* because I have not been trained in this area and because there is no available time for such cases.”*. Thirty-four participants (28.3%) described themselves as rather insecure, particularly regarding the identification of psychological crises and appropriate professional boundaries.

In contrast, another participant described feeling *“relatively confident”* in responding to students, particularly because he/she knew *“which counselling service to refer the student to.”* These largest group comprised 40 participants (33.3%) who reported feeling partially or moderately confident. These responses commonly referred to basic communication skills and knowledge of referral pathways while simultaneously emphasising limitations in formal expertise. Twenty-five participants (20.8%) described themselves as confident or very confident in dealing with students experiencing psychological crises. These respondents frequently referred to professional qualifications, prior training, or personal experiences related to mental health. An overview of the identified categories and representative quotations is presented in Table 3.

**Table 3:** Categories of perceived confidence in dealing with students experiencing psychological crises among teaching staff (*n* = 120)

Category	Description	*n*	Anchor example
Very insecure / overwhelmed	Participants described feeling highly insecure, insufficiently trained, emotionally overwhelmed, or unable to respond appropriately to psychological crises. Lack of institutional resources and uncertainty regarding responsibilities were frequently mentioned.	21	*“Very insecure*,* because I have not been trained in this area and because there is no available time for such cases.”*
Rather insecure	Respondents reported limited confidence, particularly regarding identifying crises, assessing severity, or balancing support with professional boundaries.	34	*“Insecure; even recognising psychological crises is difficult for me.”*
Partially / moderately confident	Participants described feeling somewhat prepared but emphasised situational limitations, lack of formal training, or uncertainty in more severe cases. Many referred to empathy, informal knowledge, or referral competencies rather than clinical expertise.	40	*“Relatively confident; in a conversation I could probably respond reasonably appropriately*,* and I would know which counselling service to refer the student to.”*
Confident / very confident	Participants reporting high confidence typically referred to professional qualifications, previous training, lived experience, or extensive experience working with students.	25	*“Very confident*,* because I am a psychologist and psychotherapist.”*

The table presents the results of the qualitative content analysis of free-text responses to the question assessing teaching staff’s perceived confidence in dealing with students experiencing psychological crises. Only participants actively involved in teaching activities were included in the analysis. Responses were categorized inductively based on thematic similarities. Percentages refer to the total number of valid responses (*n* = 120). Illustrative quotations were translated from German into English for reporting purposes

### Responses of teaching staff to students experiencing psychological crises

The qualitative content analysis of free-text responses regarding how teaching staff deal with students experiencing psychological crises included 124 valid responses. Because several responses contained multiple themes, responses could be assigned to more than one category. Referral to professional support services was the most frequently reported response. As one participant stated, *“I would recommend that they seek professional help and would refer them to the university psychosocial counselling service.”*. Participants described referring students to psychosocial counselling services, mental health professionals, student advisory services, or external healthcare structures. A second category concerned personal conversation and emotional support. Respondents described listening to students, offering supportive discussions, and attempting to create a supportive atmosphere. A third category referred to instrumental and academic support measures, including deadline extensions, flexible examination arrangements, online teaching options, and support with compensation measures. The identified categories and illustrative quotations are summarised in Table 4.

**Table 4:** Qualitative categories of teaching staff responses to students experiencing psychological crises (*n* = 124)

Category	Description	*n*	Anchor example
Referral to professional support services	Participants described referring students to psychosocial counselling services, mental health professionals, student advisory services, or external healthcare structures.	96	*“I would recommend that they seek professional help and would refer them to the university psychosocial counselling service.”*
Personal conversation and emotional support	Respondents emphasised listening, offering personal conversations, showing empathy, and creating a supportive atmosphere.	66	*“I always offer a personal conversation. Together*,* we try to find solutions and discuss possible ways of support.”*
Instrumental and academic support measures	Participants referred to practical accommodations, including deadline extensions, flexibility regarding coursework, online teaching options, or support with compensation measures.	30	*“I try to reduce pressure as much as possible and offer flexible solutions regarding examinations and assignments.”*

The table presents the results of the qualitative content analysis of free-text responses to the question assessing how teaching staff deal with students experiencing psychological crises. Only participants actively involved in teaching activities were included in the analysis (*n* = 124). Responses could be assigned to multiple categories; therefore, category frequencies do not sum to the total number of valid responses. Illustrative quotations were translated from German into English for reporting purposes

### Support needs of teaching staff in dealing with students experiencing psychological crises

Qualitative content analysis was additionally conducted for responses assessing perceived support needs among teaching staff in dealing with students experiencing psychological crises. A total of 128 responses were included in the analysis. Fourteen categories were identified through inductive category formation.

The most frequently reported support need concerned training, education, and professional development related to mental health and crisis management. Participants referred to workshops, continuing education opportunities, and structured training programmes. Respondents additionally emphasised the need for institutional structures and designated contact points, including clearly defined responsibilities and trained contact persons within faculties or universities. For example, one respondent stated that *“direct contacts at the faculty level who are trained”* would be helpful.

Another recurring theme concerned improved information dissemination and visibility of available support services. Several participants also referred to uncertainty regarding responsibilities, limited time resources, and role-related boundaries when dealing with students experiencing psychological crises. Additional categories included requests for procedural guidance, flexible academic regulations, crisis response systems, peer exchange opportunities, and broader structural changes within higher education institutions. The identified categories and representative quotations are presented in Table 5.

**Table 5:** Categories of perceived support needs among teaching staff in dealing with students experiencing psychological crises

Category	Description	*n*	Anchor example
No perceived need / no relevance	Respondents explicitly report no need for additional support or no relevant experience with student crises.	7	*“No need at the moment.”*
Uncertainty / lack of awareness of role or situation	Respondents express uncertainty about responsibilities or lack of experience/knowledge regarding student mental health crises.	14	*“I’m unsure whether I can help students in crisis myself.”*
Training, education, and professional development	Requests for structured training, workshops, MHFA courses, or continuing education on mental health and crisis response.	21	*“Overall*,* mandatory training sessions and regular refresher courses would be useful.”*
Guidelines, checklists, and procedural clarity	Need for structured protocols, decision aids, and step-by-step guidance for handling crisis situations.	5	*“A concrete process from the crisis to referral [help services] would be helpful.”*
Institutional structures and designated contact points	Desire for clear institutional responsibility structures, named contact persons, and formal support systems.	17	*“Direct contacts at the faculty level who are trained.”*
Low-threshold student support and counseling services	Calls for accessible, preventive, and ongoing psychosocial or counseling services for students.	12	*“More professional counseling services and low-threshold support would be important.”*
Information dissemination and visibility of services	Emphasis on better communication, visibility, and transparency of existing support services.	17	*“Better transparency of the available services and greater visibility of support.”*
Flexibility in academic processes and accommodations	Requests for flexible exam regulations, individualized accommodations, and procedural adjustments.	7	*“More flexibility in exam formats and scheduling.”*
Time constraints and workload limitations among staff	Perceived lack of time and resources to engage adequately with student mental health needs.	6	*“Chronic lack of time is a major problem.”*
Early recognition and identification of crisis signs	Need for training or tools to detect early warning signs of psychological distress.	2	*“A checklist for the early identification of crises would be helpful.”*
Crisis response systems and emergency accessibility (e.g., hotlines)	Requests for 24/7 services, emergency contacts, and immediate crisis intervention pathways.	6	*“A crisis hotline that is available at all times would be necessary.”*
Peer exchange and collaborative support structures	Desire for collegial exchange, supervision, or peer-based reflection formats.	1	*“Peer exchange meetings [intervision] would help.”*
Structural and cultural change (including stigma and responsibility shifting)	Broader reflections on institutional culture, stigma, workload distribution, and systemic reform.	6	*“There needs to be more discussion about structural changes and systemic burdens.”*
Rejection of responsibility / distancing from role	Statements rejecting responsibility or delegating responsibility entirely to external services.	5	*“I do not see this as the responsibility of the teaching staff.”*

The table presents the results of the qualitative content analysis of free-text responses to the question of perceived support needs among teaching staff in dealing with students experiencing psychological crises. Only participants actively involved in teaching activities were included in the analysis. Responses were categorized inductively based on thematic similarities. Percentages refer to the total number of valid responses (*n* = 128). Illustrative quotations were translated from German into English for reporting purposes

### Stigma-related attitudes and perceived public stigma among teaching staff

A one-way analysis of variance (ANOVA) using university affiliation as grouping variable revealed no significant differences between participating universities for either perceived public stigma (SSMIS-SF Awareness), *F*(3, 131) = 0.23, *p* = .879, η² = 0.005, or personal agreement with stigma-related stereotypes (SSMIS-SF Agreement), *F*(3, 131) = 0.12, *p* = .946, η² = 0.003.

Descriptive analyses demonstrated higher scores for perceived public stigma compared with personal endorsement of stigma-related beliefs. For the SSMIS-SF Awareness subscale, the highest mean values were observed for the items stating that people with mental illness cannot obtain or maintain regular employment (*M* = 2.25), are unpredictable (*M* = 2.23), and cannot take care of themselves (*M* = 2.09).

For the SSMIS-SF Agreement subscale, mean values were substantially lower across all items. Slight agreement with stereotype-related statements was reported most frequently for the items stating that people with mental illness are disgusting (8.3%), cannot maintain regular employment (3.8%), are dirty or unkempt (6.2%), and are unpredictable (4.6%).

**Fig. 4:**
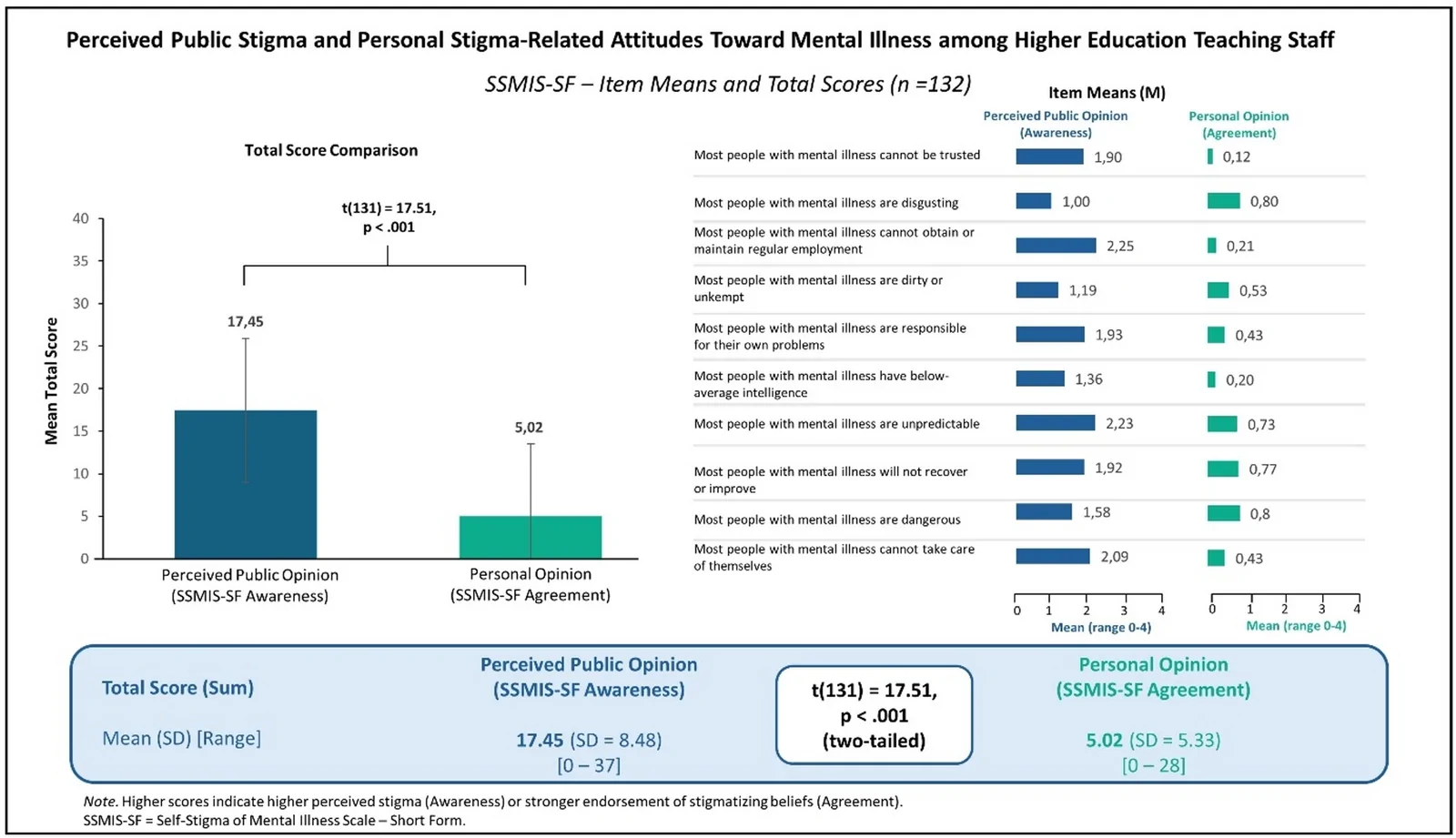
Mean scores of the SSMIS-SF subscales assessing stigma-related attitudes towards mental illness among teaching staff

A total of 132 complete cases were included in the paired analysis. Mean scores indicated higher stigma-related awareness (*M* = 17.45, *SD* = 8.48, range = 0–37) compared to agreement (*M* = 5.02, *SD* = 5.33, range = 0–28). The paired-samples t-test revealed a statistically significant difference between subscales (mean difference = 12.42, 95% CI 11.02–13.83; *t*(131) = 17.51, *p* < .001; Cohen’s *d* = 1.52, 95% CI 1.27–1.77), with awareness scores being significantly higher than agreement scores. Detailed descriptive statistics for both SSMIS-SF subscales are presented in Fig. 4.

## Discussion

The present mixed-methods study examined students’ and teaching staff’s perceptions of the role of university teaching staff in relation to student mental health promotion, psychological crisis management, and stigma-related processes within higher education settings. By integrating qualitative student perspectives with quantitative and qualitative data from teaching staff, the findings provide insights into how students and teaching staff perceive the potential gatekeeper role of teaching staff and describe mental health-related support practices.

A central finding of the study concerns the discrepancy between students’ expectations and teaching staff’s perceived professional responsibilities regarding student mental health. Students described strong expectations towards teaching staff and university institutions, emphasising the importance of psychologically sensitive communication, openness towards mental health, accessible support structures, and destigmatising learning environments. In contrast, only a minority of teaching staff perceived mental health promotion as an explicit part of their professional role. This finding suggests that student expectations and institutional role definitions may currently be insufficiently aligned within higher education contexts.

At the same time, the qualitative findings indicate that many teaching staff members nevertheless engage in substantial informal mental health-related support activities. Participants described providing emotional support, adapting teaching and assessment structures, referring students to counselling services, and creating supportive learning climates. These findings are consistent with previous literature describing university teaching staff as important “gatekeepers” who may facilitate recognition of psychological distress and promote access to professional support services [16, 21]. In the present study, however, the findings relate to perceived roles and self-reported support practices rather than objective evaluations of gatekeeper effectiveness. The results further suggest that mental health promotion in higher education is currently often based on individual commitment and relational practices rather than on clearly institutionalised responsibilities and formalised support systems. Specifically, structural approaches that affect the entire university, as opposed to individual approaches, are increasingly regarded as best practice in mental health promotion at the university [30].

Another important finding concerns the considerable heterogeneity in teaching staff’s perceived preparedness for dealing with students experiencing psychological crises. While some participants reported high levels of confidence based on professional qualifications, previous training, or personal lived experience, a substantial proportion described uncertainty, insufficient competencies, and insecurity regarding appropriate responses and professional boundaries. Across qualitative responses, many participants emphasised the absence of structured institutional training and clear procedural guidance. These findings correspond with previous research indicating that teaching staff frequently feel insufficiently prepared to respond to student mental health problems despite regularly encountering students in psychological distress [16, 21].

The findings additionally highlight the importance of institutional support structures in enabling teaching staff to respond appropriately to student mental health needs. Respondents repeatedly emphasised the need for visible counselling services, low-threshold support systems, crisis pathways, and clearly defined referral procedures. Several participants conceptualised their role not as providing direct psychological treatment, but rather as recognising distress, initiating supportive communication, and directing students towards professional services. This gatekeeper-oriented understanding of the teaching role may represent an important and realistic approach for higher education institutions, as it acknowledges both the supportive potential and the professional limitations of teaching staff.

The analyses of stigma-related attitudes revealed comparatively low levels of personal endorsement of stigmatising beliefs towards individuals with mental illness among teaching staff. However, participants simultaneously perceived stigma to be substantially more prevalent within society more broadly. These findings suggest a notable divergence between recognition of stigmatising beliefs and personal endorsement among the participating teaching staff. This discrepancy between personal attitudes and perceived public stigma is noteworthy, as perceived social norms may nonetheless influence communication behaviours, disclosure processes, and expectations regarding help-seeking. A recent study showed that stigmatising attitudes are on the rise even within the healthcare system because foundational attitudes are not addressed during medical school. The study indicates that stigmatising attitudes do not first emerge in clinical practice, but are established early in medical school and are reinforced by inadequate teaching, a lack of reflection, and insufficient practical training [31]. Previous research has similarly demonstrated that perceived stigma may remain a major barrier to support utilisation, even in contexts where explicit personal stigma appears relatively low [14, 15].

The qualitative findings from both students and staff further underline the relevance of psychologically informed and destigmatising communication within higher education. Students emphasised the importance of respectful interaction, openness regarding stress and psychological crises, and less hierarchical learning environments. Similarly, some teaching staff described role modelling, empathy, and normalisation of psychological strain as part of their professional practice. These findings align with theoretical perspectives on observational learning and role modelling, suggesting that students may be influenced not only by formal educational content but also by the interpersonal behaviours and communication styles demonstrated by teaching staff [18]. In this context, psychologically safe and inclusive learning environments may contribute to reducing barriers to disclosure and help-seeking.

From a public health perspective, the findings have several implications for higher education institutions. First, universities should strengthen institutional mental health strategies by integrating structured training opportunities for teaching staff focusing on mental health literacy, crisis recognition, communication skills, and referral procedures. Second, institutions should establish transparent and accessible support systems that reduce role ambiguity and facilitate collaboration between teaching staff and professional support services. Third, psychologically informed teaching practices and destigmatising communication strategies should be integrated more systematically into higher education policies and staff development programmes. Given the increasing prevalence of psychological distress among students, mental health promotion should not be regarded solely as an individual responsibility but as a broader institutional and public health task.

The study has several *strengths*. The mixed-methods design enabled the integration of complementary perspectives from both students and teaching staff and allowed for an in-depth exploration of subjective experiences alongside quantitative analyses of stigma-related attitudes. In addition, the inclusion of participants from multiple higher education institutions and different disciplinary backgrounds provided insights into the complexity of mental health support practices within different university settings.

Several *limitations* should be considered when interpreting the findings. First, the qualitative component was based on a single focus group with a limited number of participants and did not aim to achieve theoretical saturation. Consequently, additional perspectives may not have been captured. Second, participation in both study phases was voluntary, introducing the possibility of self-selection bias. In particular, teaching staff with a greater interest in student mental health may have been more likely to participate, potentially limiting the representativeness of the findings. Third, participants represented different higher education institutions and academic disciplines, which were not analysed separately because of limited subgroup sizes. Context-specific differences should therefore be interpreted with caution. Fourth, although validated instruments were used where available, several questionnaire items were developed specifically for this study to address the research questions and institutional context. Their psychometric properties have not yet been evaluated independently. Fifth, missing data were handled using analysis-specific case exclusion without imputation, resulting in varying sample sizes across analyses. Finally, the predominantly cross-sectional design precludes causal conclusions regarding the observed associations and perceptions. Longitudinal studies with larger and more diverse samples are warranted to confirm and extend these findings.

Future research should investigate how teaching staff can be effectively integrated into institutional mental health promotion strategies within higher education. In particular, longitudinal and intervention-based studies are needed to examine whether structured staff training programmes, psychologically informed teaching approaches, and anti-stigma interventions influence teaching staff preparedness, student help-seeking, and perceptions of psychological safety within academic environments. Therefore, future studies should additionally investigate potential associations between stigma-related attitudes, previous mental health training, disciplinary background, and perceived preparedness for supporting students experiencing psychological distress. Comparative international research may further clarify how institutional cultures and educational systems shape mental health-related support practices and stigma processes within universities.

## Conclusions

Teaching staff may represent important gatekeepers and role models for student mental health in higher education. The present findings suggest that many teaching staff members already engage in informal support practices and perceive student psychological distress as increasingly relevant within academic contexts. However, role ambiguity, limited institutional guidance, insufficient training opportunities, and unclear referral structures currently constrain their perceived preparedness to support students experiencing psychological crises.

From a public health perspective, universities should strengthen institution-wide mental health strategies and integrate psychologically informed teaching practices (e.g., communication styles, the impact of teaching staff’s implicit norms) more systematically into staff development and quality management structures. In particular, higher education institutions should provide structured training opportunities focusing on mental health literacy, crisis recognition, supportive communication, and referral procedures. In addition, visible and accessible support pathways may help reduce uncertainty among teaching staff and improve collaboration with professional mental health services.

Because psychologically safe and destigmatising learning environments may facilitate openness and help-seeking among students, mental health promotion within universities should not be regarded solely as an individual responsibility, but rather as a broader institutional and public health task.

## Supplementary Information

Supplementary Material 1.

## Data Availability

The datasets generated and/or analysed during the current study are not publicly available due to data protection regulations but are available from the corresponding author on reasonable request.

## Abbreviations

AI: Artificial intelligence
ANOVA: Analysis of variance
AOK PLUS: A German statutory health insurance provider
ISAP: Institute of social medicine, occupational health and public health
M: Mean
MAXQDA: Qualitative data analysis software
SD: Standard deviation
SBK: A German statutory health insurance provider
SPSS: Statistical package for the social sciences
SSMIS-SF: Self–stigma of mental illness scale short form
