# Supplementary material for: Teaching staff as gatekeepers for student mental health in higher education: findings from an exploratory sequential mixed-methods study on mental health promotion, psychological crises, and stigma
Source: Int J Ment Health Syst. 2026 Sep 18;20:33. doi: 10.1186/s13033-026-00738-5 (PMC13591750; doi:10.1186/s13033-026-00738-5)
Supplement: Supplementary file 1 [file 13033_2026_738_MOESM1_ESM.pdf]

UNIVERSITÄT LEIPZIG

„Studying and Staying Mentally Healthy: Universities in Dialogue“

- Survey for University Staff -

Thank you very much for taking the time to participate in our survey on promoting mental health at universities!
Please go through the questions one by one and complete the entire survey based **on your personal perspective.**
Unless otherwise indicated, please select only one option.

Generally, you should provide your answers by:
checking the box: ⊗
or entering a number or text in the appropriate lines.

1. General Information

1	How old are you?		___________ years	
2	How long have you been working at this university?		___________ years	
3	How long have you generally been working in higher education?		___________ years	
4	What is your gender?	O_0 female	O_1 male	O_2 diverse

5	In which area do you work at your university? (Multiple answers are allowed)		
	5.1 O_1 Student Services	5.6 O_1 Executive Management	5.11 O_1 Teaching & Research
	5.2 O_1 Examination Office	5.7 O_1 Property Management & Maintenance	5.12 O_1 Psychosocial Counseling
	5.3 O_1 BAföG-Office	5.8 O_1 Library & Information Management	5.13 O_1 Inclusion/Equality Officer
	5.4 O_1 Central Student Advising Office	5.9 O_1 Fundraising & Alumni Relations	5.14 O_1 Another one, namely:
	5.5 O_1 University Sports & Recreation	5.10 O_1 Administration (Finance, IT)	__________________

2. University and Mental Health Promotion

6.1	Do you see your role at the university as involving efforts to promote students' mental health?	
	O_1Yes (*continue at 6.2*)	O_0 No (*continue at 7*)
6.2	If so, please briefly describe the role you see yourself playing (based on your professional position) in promoting students' mental health (e.g., mentor, educator, support provider).	
	[Add free text here]	

3. Personal Knowledge and Skills

7	How confident do you feel when interacting with students who are experiencing a mental health crisis?
	[Add free text here]
8	How do you deal with students who are experiencing a mental health crisis, or how would you deal with them?
	[Add free text here]
9	In what areas would you like to receive support in dealing with students experiencing mental health crises?
	[Add free text here]

4. Attitudes and Stigmatization

There are many different views on people with mental illnesses. We'd like to know what you think the majority of the population or most people believe.

[continue on the next side]

UNIVERSITÄT LEIPZIG

10 Please answer the following questions using the 5-point scale provided below. Please check the box that indicates how strongly you agree or disagree. Please answer every question; do not skip any. [SSMIS-SF1]					
**I think the public believes…**	*Strongly disagree*		*neither*		*Strongly agree*
10.1. …most people with mental illness cannot be trusted.	O_0	O_1	O_2	O_3	O_4
10.2. … most people with mental illness are disgusting.	O_0	O_1	O_2	O_3	O_4
10.3. …most people with mental illness are unable to find or keep a steady job.	O_0	O_1	O_2	O_3	O_4
10.4. …most people with mental illness are dirty and unkempt.	O_0	O_1	O_2	O_3	O_4
10.5. …most people with mental illness are to blame for their problems.	O_0	O_1	O_2	O_3	O_4
10.6. …most people with mental illness have below-average intelligence.	O_0	O_1	O_2	O_3	O_4
10.7. …most people with mental illness are unpredictable.	O_0	O_1	O_2	O_3	O_4
10.8. …most people with mental illness will not recover or get better	O_0	O_1	O_2	O_3	O_4
10.9. …most people with mental illness are dangerous.	O_0	O_1	O_2	O_3	O_4
10.10. …most people with mental illness are unable to take care of themselves.	O_0	O_1	O_2	O_3	O_4

11 Now that we've asked what others might think, we'd like to know what you think. We're interested in which statements you agree with and which you disagree with. Please answer the following questions again on a 5-point scale. Please answer every question and don't skip any. [SSMIS-SF2]					
**I think…**	*Strongly disagree*		*neither*		*Strongly agree*
11.1. …most people with mental illness are to blame for their problems.	O_0	O_1	O_2	O_3	O_4
11.2. …most people with mental illness are unpredictable.	O_0	O_1	O_2	O_3	O_4
11.3. …most people with mental illness will not recover or get better.	O_0	O_1	O_2	O_3	O_4
11.4. …most people with mental illness are unable to find or keep a steady job.	O_0	O_1	O_2	O_3	O_4
11.5. …most people with mental illness are dirty and unkempt.	O_0	O_1	O_2	O_3	O_4
11.6. …most people with mental illness are dangerous.	O_0	O_1	O_2	O_3	O_4
11.7. …most people with mental illness cannot be trusted.	O_0	O_1	O_2	O_3	O_4
11.8. …most people with mental illness have below-average intelligence.	O_0	O_1	O_2	O_3	O_4
11.9. …most people with mental illness are unable to take care of themselves.	O_0	O_1	O_2	O_3	O_4
11.10. …most people with mental illness are despicable.	O_0	O_1	O_2	O_3	O_4

Thank you very much for completing the survey!
